# Item Profile Supplement Summary Score Information in Short Oral Health-Related Quality of Life Instruments

**DOI:** 10.3390/dj13100444

**Published:** 2025-09-28

**Authors:** Beáta Benke, Mike T. John, András Szentpétery, Gyula Marada

**Affiliations:** 1 Department of Prosthodontics, School of Dentistry, University of Pécs, 7625 Pécs, Hungary; benke.beata@prte.hu; 2 Department of Diagnostic and Biological Sciences, School of Dentistry, University of Minnesota, Minneapolis, MN 55455, USA; johnx055@umn.edu; 3 Department of Prosthodontics, Faculty of Dentistry, University of Szeged, 6720 Szeged, Hungary; andras.szentpetery@web.de

**Keywords:** OHIP-5, OHIP-14, oral health-related quality of life, patient-reported outcomes, psychometrics, item profiles

## Abstract

**Background:** Oral health-related quality of life (OHRQoL) questionnaires characterize the impact of oral conditions. However, similar summary scores of abbreviated instruments may obscure differences in how oral diseases affect specific OHRQoL components. **Objectives:** The aim of this study was to compare summary scores and item profiles (defined as all item prevalence scores) in two patient populations using short forms of the Oral Health Impact Profile (OHIP). **Methods**: The psychometric properties of the Hungarian OHIP-14 (14 items) and OHIP-5 (5 items) were evaluated for reliability and validity. The summary scores and item prevalence were then compared between patients undergoing surgical procedures (*n* = 30) and operative dental procedures (*n* = 22). Significant differences emerged in the OHIP-14 items “Taste worse” (9% vs. 33%, *p* = 0.03) and “Painful aching” (91% vs. 47%, *p* < 0.001). **Results**: For OHIP-5, only “Painful aching” differed significantly. Both short forms showed acceptable psychometric performance (test–retest reliability: 0.87 and 0.86; Cronbach’s alpha: 0.88 and 0.66; validity with self-reported oral health: r = 0.48 and r = 0.51). **Conclusions**: Summary scores provide an overall assessment of OHRQoL, but item profiles reveal clinically relevant differences between patient groups. Combining both perspectives enhances the interpretability of short OHIP instruments and supports more targeted clinical and research applications.

## 1. Background

Oral health-related quality of life (OHRQoL) questionnaires have gained increasing popularity in characterizing and comparing the impact of oral conditions, especially if the instruments are brief and minimize patient burden. For example, the original Oral Health Impact Profile (OHIP) [1] has 49 items, but short versions from 30 [2] to 5 items (OHIP-5) [3] exist. While a 14-item questionnaire (OHIP-14) which was originally developed as an English-language instrument by Slade [4] is the most widely used OHRQoL tool, recent recommendations for OHIP versions favor OHIP-5 because of its ability to assess the four dimensions of OHRQoL Oral Function, Orofacial Pain, Orofacial Appearance, and Psychosocial Impact [5,6] OHIP-based assessment of these dimensions is globally available for at least 75% of the most widely spoken languages [6], and it allows a standardized oral health impact assessment for all oral diseases in all settings [7].

For OHIP-based OHRQoL assessment in Hungary, a long Hungarian OHIP version (OHIP-H49) is available, and its psychometric properties have been determined [8]. OHIP-H49 items allowed the creation of OHIP-H14, the widely used version, and OHIP-H5, the shortest version.

Usually, only the summary scores of instruments are presented to characterize the construct OHRQoL as a whole. So-called “norms” enabling us to compare actual scores to population data have been developed for the UK oral health-related quality of life measure (OHRQoL-UK) [9], the German version of the OHIP [10], and the Swedish version of the OHIP [11]. However, both the “scores” and the “norms” render only short and concentrated information about the state of OHRQoL. Summary scores of similar magnitudes may hide the differential impact of oral conditions on individual components of OHRQoL when subjects with different oral conditions are compared. Item profiles (defined as all item prevalence scores for the instrument) may provide additional information above and beyond summary scores.

In line with recent developments in patient-reported outcome measurement, combining summary scores with item profile analysis can offer a more nuanced view of how oral health problems affect individuals. This dual approach captures both the overall burden and the specific nature of impairments, making it particularly valuable in clinical decision-making and public health planning. Furthermore, investigating item-level differences between populations contributes to the broader debate on the balance between instrument brevity and informational richness, an issue of growing importance in health outcomes research.

The aims of this study were 1. to evaluate the psychometric properties of the two short Hungarian OHIP instruments with 14 and 5 items, respectively, and 2. to investigate the hypothesis that, in the absence of summary score differences between typical dental patient populations, differences in the prevalence of individual items on the OHIP-14 and the OHIP-5 may exist.

## 2. Methods

### 2.1. Evaluation of OHIP-H14’s and OHIP-H5’s Psychometric Properties

The items for OHIP-14 and OHIP-5 were taken from the long Hungarian OHIP (OHIP-H49) [8] according to the 14-item English [4] and the 5-item German [3] OHIP instruments. For the OHIP questions, subjects were asked how frequently they had experienced the impact of that item in the last month. Responses were based on a scale of 0—never; 1—hardly ever; 2—occasionally; 3—fairly often; and 4—very often. For a comparison of item prevalence, “any impact” was defined as response codes “hardly ever” to “very often”. Summary scores were derived for the long Hungarian form (OHIP-H49) and the two short forms (OHIP-H14 and OHIP-H5—see the Appendix A) by computing a simple sum of item responses. (The convention for naming the questionnaires is similar to that in the case of the German OHIP instruments: for example, H and G stand for the country (Hungary or Germany), respectively, and 14 and 5 indicate the numbers of included items.)

Reliability was evaluated by studying the OHIP scores’ internal consistency and temporal stability. For the former, in 200 randomly selected general population subjects (mean age: 48.8 ± 17.2 years, 63% women) (the subject recruitment was as described earlier [7]), the Cronbach’s alpha [12] and average interitem correlation were computed. For the latter, the test–retest reliability was determined in a convenience sample of 42 prosthodontic patients (mean age: 51.9 ± 16.9 years, 64.3% women) before treatment using a time interval of two weeks between two OHIP administrations. It was assumed that no substantial change in OHIP summary scores happened between the two administration times. Intraclass correlation coefficients based on one-way repeated measures analysis of variance were calculated for the OHIP summary scores. Limits of agreement [13] were computed around the mean differences (Time1 minus Time2). Guidelines were applied to judge the magnitude of the test–retest reliability coefficients [14] and for Cronbach’s alpha [15,16].

Construct validity was tested in a convenience sample of 112 consecutive general dentistry patients (mean age: 43.9 ± 18.9 years, 54.5% women) seen at the Dental School of the University of Pécs, Hungary. The correlation coefficients between the short-form summary scores and the long OHIP summary score (Pearson correlation coefficient), as well as between both long- and short-form OHIP summary scores and self-reported oral health (Spearman rank correlation), were computed for validity assessment.

To improve transparency and reproducibility, the methods followed international recommendations for psychometric studies, including the COSMIN checklist. The translation and cultural adaptation of the Hungarian OHIP followed forward–backward translation procedures and was piloted before final data collection. All questionnaires were self-administered in a quiet clinical environment, with trained assistants available to clarify wording if needed. To minimize potential bias, instructions were standardized, and the order of items was identical for all participants.

### 2.2. Study Participants

The study subjects came from two typical dental patient populations at the School of Dentistry, University of Pécs, Hungary, specifically patients undergoing surgical procedures (third molar extractions) (*n* = 30, mean age: 45.2 ± 17.6 years, 47% women) or operative dental procedures (*n* = 22, mean age: 31.3 ± 11.8 years, 52% women). In the operative dentistry group, all patients received Class I or Class II composite restorations in premolar or molar teeth. The location within the maxilla or mandible was not systematically recorded. The surgical dental treatment group consisted exclusively of patients undergoing third molar extractions. In this dataset, we did not systematically distinguish between mandibular and maxillary extractions, although clinical experience suggests that mandibular extractions were more common. Future studies should record this detail to allow a more nuanced analysis of potential quality of life differences between maxillary and mandibular cases. Two of the OHIP-14 item prevalence scores were significantly different. The participants were a convenience sample. All participants consented in filling out the questionnaire and filled out the OHIP-H49 questionnaire.

The eligibility criteria were predefined: The inclusion criteria were age ≥ 18 years, the ability to understand Hungarian, and willingness to participate. The exclusion criteria included acute systemic illness, previous oral surgery in the last four weeks, cognitive impairment, or refusal to provide informed consent. Recruitment was consecutive, meaning that every eligible patient attending the dental school within the study period was invited to participate until the target numbers were reached. Ethical approval was obtained from the Medical Faculty of the University of Pécs (protocol number 2421—PTE 2005, updated 2421–PTE, approved on 6 February 2024). Because orofacial pain is a leading reason for seeking dental treatment and substantially affects OHRQoL, we emphasize this dimension in our analyses [17,18,19].

### 2.3. Data Analysis

Summary scores from the two short OHRQoL instruments (OHIP-H14 and OHIP-H5) for the two patient populations were compared using t-tests. Differences in item prevalence (representing any OHRQoL impact) were evaluated using two-sample tests of proportions. Subjects (*n* = 3) were not included in the analyses if data was missing to the extent that the amount missing compromised the calculation of the long OHIP summary score. These three subjects were excluded from the study. For subjects having less missing data than the specified cutoffs, the missing values were imputed using a regression procedure [20]. The Ethics Committee of the Medical Faculty of the University of Pécs approved the study. All patients were asked to give their informed consent at the time of completion of the questionnaire.

Statistical analyses were performed using IBM SPSS Statistics for Windows, Version 24.0 (IBM Corp., Armonk, NY, USA). Parametric assumptions were checked prior to applying t-tests. Where applicable, effect sizes were calculated (Cohen’s d for continuous outcomes, Cohen’s h for proportions) to supplement *p*-values. Internal consistency was judged according to widely accepted benchmarks (Cronbach’s alpha ≥ 0.70 considered acceptable). The test–retest reliability was interpreted using ICC thresholds (≥0.75 good, ≥0.90 excellent). Confidence intervals are reported alongside point estimates to provide a measure of precision. Sensitivity analyses were carried out to test the robustness of the results, for example, by excluding imputed cases [21].

## 3. Results

### 3.1. Psychometric Properties of the Abbreviated Instruments

Cronbach’s alpha (0.88) was “satisfactory” according to guidelines, and the interitem correlation (0.35) provided additional evidence for the internal consistency of the OHIP-H14 scores (Table 1). The coefficient alpha for OHIP-H5 (0.66) was close to “satisfactory” reliability, but the interitem correlation (0.28) was only slightly lower than that in the 14-item instrument. The test–retest reliability coefficients (intraclass correlation coefficients—ICCs: 0.87 and 0.86) were “excellent” according to the guidelines for both instrument scores. The mean OHIP summary score differences between the two assessments were small (−0.6 OHIP-H14 and 0.3 OHIP-H5 points) and indicated no change in the construct OHRQoL. The limits of agreement (−10.3 to 9.1 and −3.7 to 4.3 for OHIP-H14 and OHIP-H5, respectively) indicated acceptable variability for both instruments’ individual score differences. The correlations between the abbreviated instruments’ summary scores and the OHIP-H49 summary score (OHIP-H14: r_Pearson_ = 0.96; OHIP-H5: r_Pearson_ = 0.90) or the long instrument’s summary score excluding the 5 or 14 items (both r_Pearson_ = 0.86) supported the short instruments’ validity. The self-reported oral health and OHIP summary scores were substantially correlated (Spearman rank correlations: 0.48 for OHIP-H14, and 0.51 for OHIP-H5) in dental patients (Table 1).

The internal consistency indices (Cronbach’s alpha and interitem correlations) suggest that the OHIP-H14 provides a more homogeneous measurement of the construct than the OHIP-H5, although both instruments achieved acceptable reliability. The test–retest results demonstrated high temporal stability, confirming that both questionnaires yield reproducible outcomes over short time intervals. No major floor or ceiling effects were observed, indicating that both short forms were able to capture the full range of possible responses without clustering at the extremes.

### 3.2. OHRQoL Summary Scores and Item Profiles in Patients Undergoing Surgical or Operative Dental Procedures

**Figure 1:**
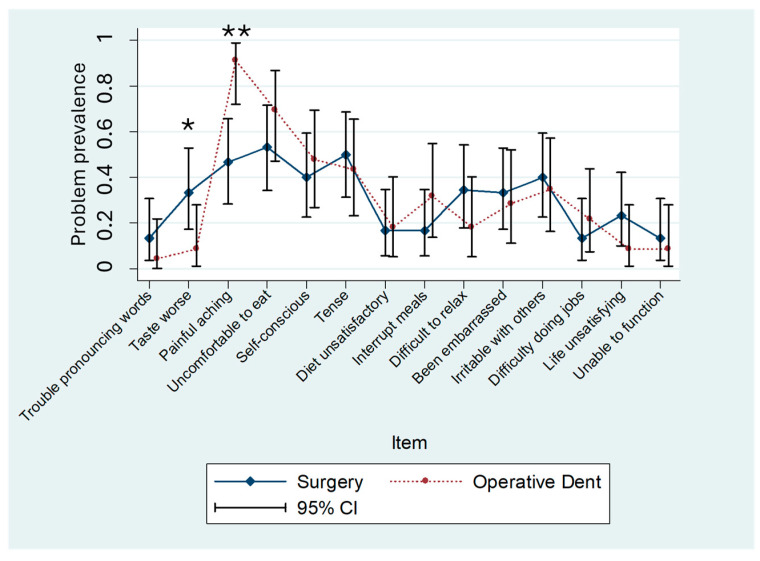
OHIP-H14 item prevalence for oral surgery or operative dentistry patients. Prevalence and 95% confidence intervals (CIs) for oral surgery patients (*n* = 30) and operative dentistry patients (*n* = 22). Group differences indicated with asterisks (* item number 2, “Taste worse,” *p* = 0.03, and ** item number 3, “Painful aching,” *p* < 0.001).

**Figure 2:**
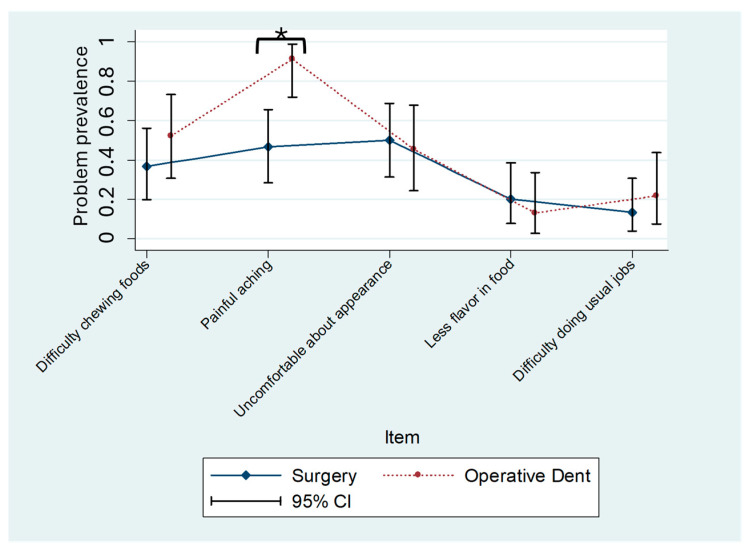
OHIP-H5 item prevalence for oral surgery or operative dentistry patients. Prevalence and 95% confidence intervals (CIs) for oral surgery patients (*n* = 30) and operative dentistry patients (*n* = 22). Group differences indicated with asterisk (* item number 2, “Painful aching,” *p* = 0.005).

The OHIP-H14 summary scores were similar in both patient populations (8.7 ± 9.3 vs. 8.3 ± 7.3 OHIP points; mean ± standard deviation). The OHIP-H5 summary scores were slightly more different (3.9 ± 3.8 vs. 5.0 ± 3.3 OHIP points) between the two groups. The differences in mean scores between patient groups for both instruments were statistically nonsignificant (OHIP-H14: *p* = 0.87; OHIP-H5: *p* = 0.30). However, two of the OHIP-H14 item prevalence scores were significantly different in the patient populations (item number 2, “Taste worse,” *p* = 0.03, and item number 3, “Painful aching,” *p* < 0.001) (Figure 1). In patients undergoing operative dental procedures, “Taste worse” was reported by 9% of the population; however, 33% of patients undergoing oral surgery reported the same problem. For the prevalence of “Painful aching”, the magnitude of the problem was higher in operative dentistry patients (91%) compared to oral surgery patients (47%). The prevalence of only one item was different in the two patient populations for OHIP-H5 (“Painful aching”—the identical item contained in OHIP-14) (Figure 2).

Although the overall summary scores did not differ significantly between groups, item-level analysis revealed meaningful clinical contrasts. The higher prevalence of “Taste worse” among oral surgery patients is consistent with postoperative sensory disturbances, while the increased reporting of “Painful aching” in operative dentistry patients likely reflects acute pain following restorative interventions. These item-specific patterns, highlighted by the OHIP-H14, demonstrate the added diagnostic value of item profiles in distinguishing between patient populations with otherwise comparable total scores.

Beyond demonstrating psychometric validity, our findings also point to the specific items that contributed additional information beyond summary scores. In particular, the items “Taste worse” and “Painful aching” differentiated between oral surgery and operative dentistry patients, despite similar total OHIP scores. These results underline that sensory and pain-related dimensions may be especially sensitive to treatment type and clinical condition.

More generally, such item-level differences emphasize that not all OHIP questions contribute equally to distinguishing between patient populations. Pain-related items, for example, may dominate the profiles of patients undergoing operative dentistry, while sensory changes (such as altered taste) appear more relevant in oral surgery contexts. A more systematic evaluation of which items consistently add discriminative value could improve both the design of short forms and their interpretation in clinical practice.

Subgroup analyses adjusting for age and sex yielded similar findings, suggesting that the observed differences are attributable primarily to treatment type rather than demographic variation. The confidence intervals for item prevalence estimates showed minimal overlap in the most divergent items, further supporting the robustness of these results.

## 4. Discussion

This study demonstrated that OHIP-H14 and OHIP-H5 had sufficient validity and reliability, and they can be used instead of the long instrument if the patient burden needs to be minimized. Similar OHRQoL impairments as characterized by the summary scores of the OHIP-14 and OHIP-5 instruments were accompanied by different OHIP item prevalences in two different patient populations, one undergoing operative dental procedures and the other undergoing surgical procedures. This phenomenon may not be limited to these specific populations and is therefore of wider interest for the use of the OHIP and other OHRQoL instruments.

The psychometric properties for OHIP-14 and OHIP-5 have been relatively comparable in different patient population settings. For example, for OHIP-5, our study results are in line with a correlation between OHIP-5 and OHIP-49 of r = 0.94 in 163 German general population subjects and r = 0.91 in 175 German TMD patients [3]. In 245 Dutch TMD patients, the correlations between the summary scores of the 5-, 14-, and 49-item OHIPs were all high and above 0.90 [18]. Comparable results have also been reported in German general population samples and Thai TMD patients [22,23]. In 1309 adult subjects from the Swedish general population, similar results were observed [11]. A correlation of r = 0.95 was found in 331 Spanish-speaking dental patients in a US health care setting [7].

Our findings extend these international results by adding data from the Hungarian context, where the availability of short OHIP forms has been limited. The strong correlations with the long OHIP version confirm that shortened instruments can be reliably applied across cultures, supporting their inclusion in multinational studies.

Among the four dimensions of OHRQoL Oral Function, Orofacial Pain, Orofacial Appearance, and Psychosocial Impact, Orofacial Pain is the most frequent reason why dental patients, adults and children alike, seek dental treatment [17,18]. However, the extent of dental and orofacial pain varies considerably among different dental patient populations. For example, for patients with temporomandibular disorders (TMDs), pain is the major complaint [19], but for prosthodontic patients with tooth loss, pain is usually less of a concern, although the overall burden from oral problems may not be too different between the two populations [20]. We did not observe statistically significant differences between OHIP descriptors of oral function in our populations. This may be due to the limited sample size in the present study. However, differences of 10–20% in cardinal oral functions such as “chewing”, “talking”, and “taste” may be important from a clinical perspective. For the psychosocial OHRQoL item, we did not observe statistically significant differences. However, in a different study comparing two populations with known substantial psychosocial burden (patients with temporomandibular disorders and patients with dental anxiety) [24], significant differences in psychosocial problem prevalence were found (e.g., “Been embarrassed”: reported by 42.2% of anxiety patients but only 4.2% of TMD patients). In the same study, the OHIP summary scores and the prevalence of OHIP-14 items in these populations with psychosocial burden also varied.

These findings suggest that, while summary scores provide a useful overview, they may obscure clinically meaningful differences at the item level. For clinicians, item profiles can highlight specific areas—such as functional limitations or pain—that require targeted intervention. For researchers, this underlines the importance of reporting both total scores and item prevalence, particularly in comparative studies.

Longitudinal studies can also provide evidence that item prevalence may yield additional information about OHRQoL above and beyond summary scores. In a previous study in prosthodontic patients [22], problems typically reported by patients before prosthetic treatment (e.g., “Difficulty chewing”, “Take longer to complete meal”, “Food catching”, “Uncomfortable to eat”, and “Unable to eat (dentures)”) disappeared 6 to 12 months after treatment. However, new (“posttreatment”) problems such as “Sore spots”, “Painful gums”, and “Discomfort (dentures)” emerged. Although OHRQoL improved in general from baseline to follow-up as indicated by lowered OHIP summary scores, the patterns of some items decreasing in their frequency and some items increasing in their frequency cannot captured by the summary score. This finding may support the importance of item profiles when assessing changes in OHRQoL. The observed differences in item prevalence in the present study are not likely due to methodological problems with the short instruments, because our reliability and validity analyses produced similar findings to those observed with other OHIP short forms [2,6,7,12,23,25].

From a methodological perspective, these results reinforce the value of combining classical psychometric testing with item-level analysis. While internal consistency and reliability indices ensure measurement quality, item prevalence patterns shed light on the specific patient experiences underlying the scores. This dual approach enhances both the scientific rigor and the clinical applicability of OHRQoL research.

This study was carried out on only two specific dental patient populations; however, it seems probable that similar item profile differences also exist between other population groups with similar summary scores. Moreover, there are no known culturally specific concepts included in OHIP-H that may restrict the broad applicability of our results towards other cultural environments.

## 5. Limitations

This study has some limitations. First, the sample size was relatively small and restricted to two dental patient populations from a single university clinic, which may limit the generalizability of the findings. Second, the cross-sectional design does not allow for the evaluation of changes in oral health-related quality of life over time. Finally, although validated translations of OHIP instruments were used, cultural factors may still influence how patients interpret individual items. These limitations should be considered when interpreting the results. Additionally, the study did not systematically record whether third molar extractions were mandibular or maxillary. This distinction may be relevant, as postoperative recovery and functional impact differ between sites. Future studies should therefore include this information to better capture potential differences in OHRQoL outcomes.

## 6. Future Directions

Future research should expand these findings by including larger and more diverse populations, ideally through multi-center and cross-national collaborations. Longitudinal designs are needed to evaluate how item profiles change over time and to capture the dynamics of oral health-related quality of life. Furthermore, incorporating qualitative methods such as interviews or focus groups may provide richer insights into how patients experience oral health impacts beyond what is measured by questionnaires.

## 7. Conclusions

The present study demonstrated that, although short versions of the OHIP (OHIP-5 and OHIP-14) yielded similar overall summary scores across patient groups, item-level analyses revealed clinically relevant differences between oral surgery and operative dentistry patients. These findings highlight that relying solely on total scores may mask important variations in the impact of oral conditions.

By combining summary scores with item profiles, clinicians can gain a more comprehensive picture of their patients’ oral health-related quality of life. This approach supports a more personalized and needs-based treatment planning. For researchers, our results confirm that shortened OHIP instruments can provide valid and reliable data without losing critical information, provided that item-level responses are also considered.

Overall, this study underscores the value of supplementing traditional summary measures with item profiles, thereby improving both the clinical applicability and research utility of OHRQoL assessment tools.

The questions of the OHIP-H5 and OHIP-H14 are presented in the Appendix A.

## Figures and Tables

**Table 1:** Psychometric properties for the OHIP-H14 and the OHIP-H5.

	Construct Validity (Dental Patients *n* = 112)	Test–Retest Reliability (Prosthodontic Patients *n* = 42)			Internal Consistency (General Population Subjects *n* = 200)	
Instrument (Number of Items)	Spearman Correlation Coefficient and Level of Statistical Significance (Correlation with Self-Reported Oral Health)	ICC (95% CI ^†^)	Mean of the Differences (95% CI ^†^)	Limits of Agreement	Cronbach’s Alpha	Average Interitem Correlation
OHIP-H14 (14)	0.48 ***	0.87 (0.79 to 0.94)	−0.6 (−2.4 to 0.9)	−10.3 to 9.1	0.88	0.35
OHIP-H5 (5)	0.51 ***	0.86 (0.79 to 0.94)	0.3 (−0.3 to 0.9)	−3.7 to 4.3	0.66	0.28

ICC = Intraclass correlation coefficients. ^†^ Confidence interval. *** *p* < 0.001.

## Data Availability

The original contributions presented in this study are included in the article. Further inquiries can be directed to the corresponding author(s).

## Abbreviations

The following abbreviations are used in this manuscript:

OHRQoL	Oral health-related quality of life
OHIP	Oral Health Impact Profile

## Appendix A

The questions of the two short instruments—OHIP-H5 and OHIP-H14:

OHIP-H5
1 Difficulty chewing
2 Painful aching
3 Appearance uncomfortable
4 Less flavor in food
5 Difficulty doing jobs

OHIP-H14
1 Trouble pronouncing words
2 Taste worse
3 Painful aching
4 Uncomfortable to eat
5 Self-conscious
6 Tense
7 Diet unsatisfactory
8 Interrupt meals
9 Difficult to relax
10 Been embarrassed
11 Irritable with others
12 Difficulty doing jobs
13 Life unsatisfying
14 Unable to function
